# Caspase activation counteracts interferon signaling after G2 checkpoint abrogation by ATR inhibition in irradiated human cancer cells

**DOI:** 10.3389/fonc.2022.981332

**Published:** 2022-10-28

**Authors:** Adrian Eek Mariampillai, Sissel Hauge, Inger Øynebråten, Gro Elise Rødland, Alexandre Corthay, Randi G. Syljuåsen

**Affiliations:** ^1^ Department of Radiation Biology, Institute for Cancer Research, Oslo University Hospital, Oslo, Norway; ^2^ Tumor Immunology Lab, Department of Pathology, Oslo University Hospital, Oslo, Norway; ^3^ Hybrid Technology Hub – Centre of Excellence, Institute of Basic Medical Sciences, University of Oslo, Oslo, Norway

**Keywords:** cell cycle checkpoints, type I interferon (IFN) signaling, radiation therapy (radiotherapy), micronuclei (MN), ATR, caspase, cGAS, TREX1

## Abstract

Recent studies suggest that inhibition of the ATR kinase can potentiate radiation-induced antitumor immune responses, but the extent and mechanisms of such responses in human cancers remain scarcely understood. We aimed to assess whether the ATR inhibitors VE822 and AZD6738, by abrogating the G2 checkpoint, increase cGAS-mediated type I IFN response after irradiation in human lung cancer and osteosarcoma cell lines. Supporting that the checkpoint may prevent IFN induction, radiation-induced IFN signaling declined when the G2 checkpoint arrest was prolonged at high radiation doses. G2 checkpoint abrogation after co-treatment with radiation and ATR inhibitors was accompanied by increased radiation-induced IFN signaling in four out of five cell lines tested. Consistent with the hypothesis that the cytosolic DNA sensor cGAS may detect DNA from ruptured micronuclei after G2 checkpoint abrogation, cGAS co-localized with micronuclei, and depletion of cGAS or STING abolished the IFN responses. Contrastingly, one lung cancer cell line showed no increase in IFN signaling despite irradiation and G2 checkpoint abrogation. This cell line showed a higher level of the exonuclease TREX1 than the other cell lines, but TREX1 depletion did not enhance IFN signaling. Rather, addition of a pan-caspase inhibitor restored the IFN response in this cell line and also increased the responses in the other cell lines. These results show that treatment-induced caspase activation can suppress the IFN response after co-treatment with radiation and ATR inhibitors. Caspase activation thus warrants further consideration as a possible predictive marker for lack of IFN signaling.

## Introduction

Local radiotherapy can increase tumor immunogenicity, yielding systemic, abscopal effects on distal metastases in rare cases (1, 2). However, the influence of radiotherapy on the immune system is complex, and radiotherapy may also stimulate immunosuppressive mechanisms (3). Immune checkpoint inhibitors combined with radiotherapy has shown promise in enhancing the antitumor immune effects (4–6). Nevertheless, therapeutic responses remain limited, urging the need for more knowledge and new, efficacious strategies.

The serine/threonine protein kinase ATR is a central regulator of the G2 cell cycle checkpoint and DNA repair following irradiation (7, 8). When ATR inhibitors (ATRi) are combined with irradiation, cells will enter mitosis with unrepaired DNA lesions, which ultimately causes micronucleus formation and cell death (9). ATRi are therefore promising radiosensitizers under clinical evaluation (10, 11). Interestingly, recent studies suggest that ATRi, besides their effects on cell cycle checkpoints and DNA repair, may also increase radiation-induced antitumor immune responses. Increased immune effects, such as activation of CD8^+^ T cells and immunological memory, have been observed in murine cancer models after treatment with the ATR inhibitor AZD6738 and ionizing radiation (IR) (12–14). Mechanistically, ATRi may stimulate tumor immunogenicity through downregulation of programmed cell death 1 ligand 1 (PD-L1) in irradiated cancer cells (3, 14, 15). In addition, ATR inhibition can potentiate radiation-induced type I IFN responses, likely through generation of cytosolic DNA resulting from increased micronucleus formation after abrogation of cell cycle checkpoints (16, 17). In this scenario, the DNA sensor cGAS recognizes *de facto* cytosolic DNA in ruptured micronuclei, and triggers induction of type I IFN through the cGAS–STING–IRF3–TBK1 signaling cascade (18–20). Noteworthy, the cGAS–STING–IFN pathway is negatively regulated by three-prime repair exonuclease 1 (TREX1), which degrades the DNA substrates of cGAS (21, 22). In addition, this pathway can be negatively regulated by caspase-mediated protein cleavage (23).

The potentiation of IFN responses after IR and ATRi were mostly shown in murine cancer or human normal cells, and it remains elusive whether similar effects commonly occur in human cancer cells. Furthermore, in some cell lines, IFN responses were rather stimulated through immune recognition of cytosolic RNA (16, 17). Opposing results regarding whether the IFN response was dependent on the cytosolic RNA sensor RIG-I or the DNA sensor cGAS have even been reported for the same cells (MCF10A) (16, 17), underlining the mechanistic uncertainty of the response.

Here, we investigated the hypothesis that combined treatment of human cancer cells with IR and ATRi stimulates cGAS-mediated type I IFN responses, due to G2 checkpoint abrogation and consequently enhanced generation of micronuclei. We found that the combined treatment caused increased cGAS-mediated type I IFN secretion in all tested cell lines except for one, which contained very high basal levels of the exonuclease TREX1. However, downregulation of TREX1 in this cell line did not restore IFN signaling. Rather, the IFN response was restored upon co-treatment with a pan-caspase inhibitor. The caspase inhibitor also further increased the IFN responses in the other cell lines.

## Results

### Radiation-induced type I interferon signaling declines at high radiation doses, coinciding with a prolonged G2 checkpoint arrest

**Figure 1:**
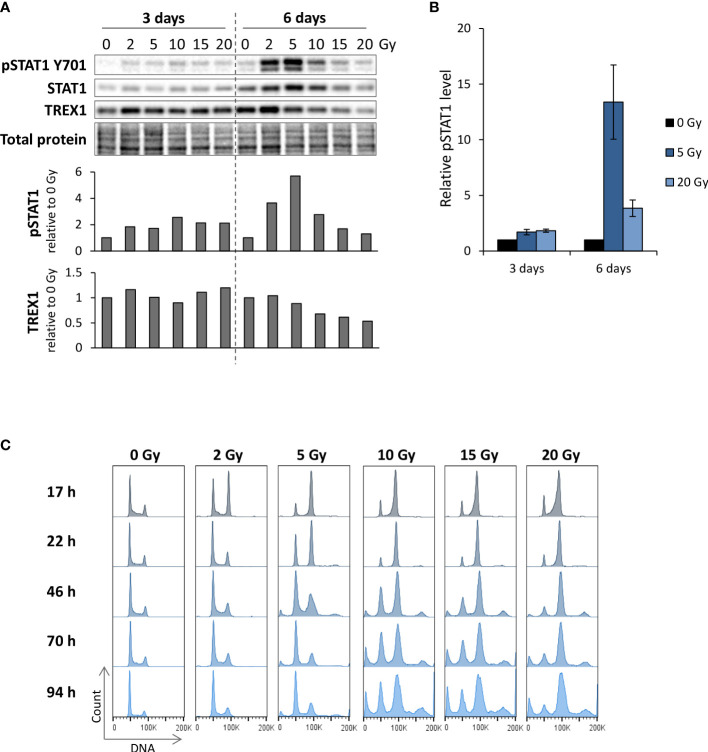
Reduction of radiation-induced IFN signaling at high IR doses coincides with prolonged G2 checkpoint arrest. **(A)** Immunoblots of U2OS cells harvested at three and six days after IR. Bar charts show pSTAT1 and TREX1 levels relative to total protein and normalized to mock. **(B)** Quantification of pSTAT1 levels relative to the corresponding mock sample for multiple independent experiments with 5 and 20 Gy as in **(A)**. (3 days: *n* = 5 for 5 Gy and *n* = 4 for 20 Gy; 6 days: *n* = 3) **(C)** DNA histograms from parallel samples in the same experiment as in **(A)**. The ‘100K’ annotation marks the G2/M phase peak. Results in **(A, C)** are representative for three independent experiments performed at different time points within 0-6 days post treatment.

To explore how ATR inhibitors affect radiation-induced IFN signaling, we first assessed the effects of irradiation alone. We treated the human osteosarcoma cell line U2OS with different radiation doses (2-20 Gy), and measured IFN signaling three to six days post treatment by immunoblotting of phosphorylated STAT1 (pSTAT1). STAT1 is phosphorylated upon autocrine and paracrine type I IFN signaling, rendering pSTAT1 indicative of IFN secretion (18, 24). At six days post treatment, a marked increase in pSTAT1 was observed after lower radiation doses (2 and 5 Gy), whereas higher doses (>10 Gy) gave only minor increases in pSTAT1 level (**Figures 1A, B**). Similar radiation dose responses have been reported in a previous study, where the lack of IFN secretion after higher doses (> 10 Gy) was attributed to radiation-induced increases in *TREX1* expression (25). Contrastingly, we did not find any increase in TREX1 levels in U2OS cells after irradiation with 10-20 Gy (**Figure 1A**). Our results thus suggest other mechanisms to be responsible for suppression of IFN responses after high-dose irradiation in this cell line. Induction of type I IFN responses has been linked to formation of micronuclei resulting from mitosis with unrepaired DNA after irradiation (18, 19). As arrest at the G2 checkpoint delays mitotic entry, we compared cell cycle progression after low- and high-dose irradiation. The cells arrested notably longer in the radiation-induced G2 checkpoint after higher doses than after lower doses, as expected (**Figure 1C**). The lack of IFN signaling after exposure to high doses of radiation thus coincides with prolonged G2 checkpoint arrest, suggesting that the arrest counteracts IFN signaling.

### ATR inhibition-induced G2 checkpoint abrogation accelerates micronucleus formation after irradiation

**Figure 2:**
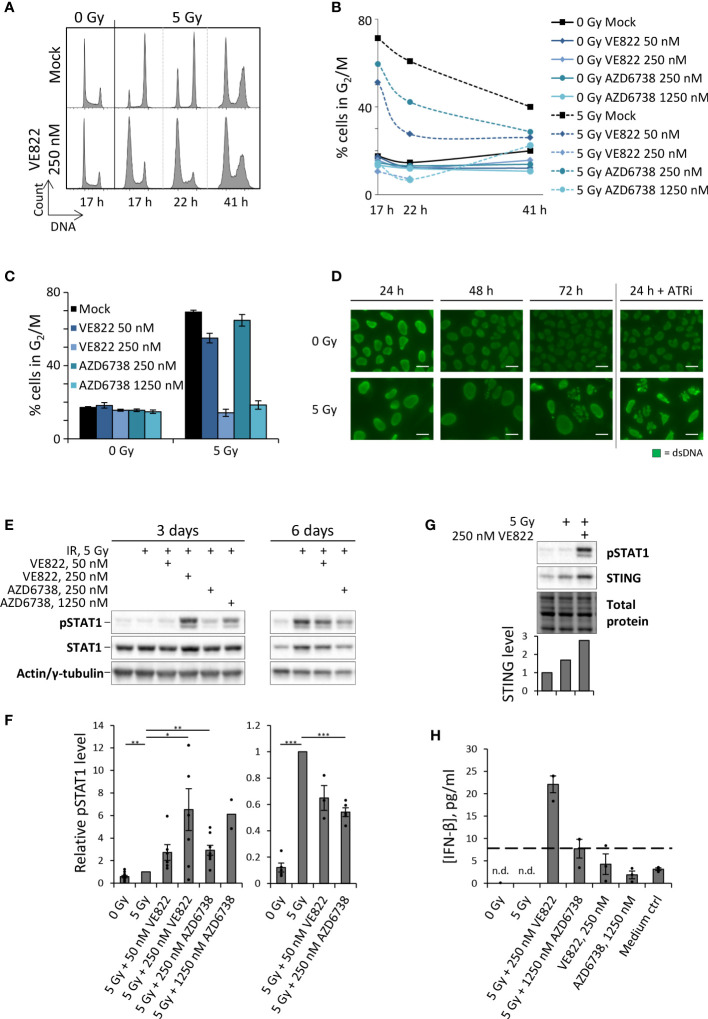
ATRi abrogates radiation-induced G2 checkpoint arrest, resulting in expedited generation of micronuclei and induction of type I IFN response in U2OS cells. **(A)** DNA histograms after treatment with IR and VE822. **(B)** Quantification of proportion of cells in G2/M phase from the experiment in **(A)**. **(C)** Bar-plotted quantification of G2/M proportions from three independent experiments performed as in **(A)**, at 17 hours after treatments. **(D)** Micrographs showing anti-dsDNA immunofluorescence staining. ATRi: 250 nM VE822. Scale bars: 20 µm. **(E)** Immunoblot of phosphorylated STAT1 (pSTAT1) and total STAT1 (STAT1) three and six days after IR with or without VE822 and AZD6738. Pan-actin and γ-tubulin were used as loading controls at three and six days, respectively. **(F)** Quantification of pSTAT1 levels relative to loading controls for experiments as in **(E)**. Values are normalized to 5 Gy. **(G)** Immunoblot of pSTAT1 and STING in U2OS cells at three days after the indicated treatments. Bar chart shows STING level relative to total protein and normalized to mock. **(H)** ELISA of IFN-β in 20X up-concentrated growth media from U2OS cells harvested three days after treatment. Dashed line indicates the lowest interferon-β concentration tested in the standard curve in **Supplementary Figure S1E** (7.81 pg/ml). n.d. = not detectable.

We next investigated whether ATR inhibition can abrogate the G2 checkpoint after irradiation with low and high doses. We employed the ATR inhibitor VE822 (berzosertib) at a high concentration (250 nM), which caused ~80% reduction in cell viability (**Supplementary Figure S1A**, left). Treatment with 250 nM VE822 efficiently abrogated the checkpoint after 2 and 5 Gy irradiation, but less so after irradiation with 10 or 20 Gy (**Supplementary Figure S1B**). Hence, ATR inhibition is less effective in abrogating G2 checkpoint arrest after higher radiation doses, in agreement with previous studies showing that the G2 checkpoint is regulated by multiple factors (26–30). In our subsequent studies with radiation and ATRi, we therefore irradiated with 5 Gy. U2OS cells showed a pronounced G2 checkpoint arrest at 17 hours after 5 Gy, with the cell cycle profile slowly beginning to redistribute at 22-41 hours post treatment (**Figure 2A**). Cells co-treated with 5 Gy and 250 nM VE822 showed no sign of checkpoint arrest, with no accumulation of cells in G2 phase at 17-22 hours post treatment (**Figure 2A**). Furthermore, the G2 checkpoint was almost completely abrogated at 0-6 hours post treatment, as detected by presence of mitotic cells (data not shown). The checkpoint was correspondingly abrogated by a high concentration (1250 nM) of the ATR inhibitor AZD6738 (ceralasertib) (**Figures 2B, C**). This concentration of AZD6738 caused ~50% reduction in cell viability (**Supplementary Figure S1A**, right). We also tested lower, less toxic concentrations of both VE822 and AZD6738 (50 nM and 250 nM, respectively), yielding 5-10% reduction in viability (**Supplementary Figure S1A**). The lower concentrations gave a partial abrogation of the G2 checkpoint (**Figures 2B, C**). The effect of ATR inhibition was also assessed by immunofluorescence microscopy. Cells treated with 5 Gy IR and 250 nM VE822 generated micronuclei already within 24 hours post treatment (consistent with finalized mitosis), whereas micronuclei were observed at 72 hour post treatment for irradiated mock cells (**Figure 2D**). These results indicate that ATR inhibitors at high concentrations efficiently abrogate G2 checkpoint arrest and thereby accelerate the generation of micronuclei.

### Combined treatment with IR and ATRi expedites radiation-induced interferon response in U2OS cells

Type I IFN responses upon treatment of U2OS cells with IR and ATRi were measured by pSTAT1 levels and IFN-β ELISA. Irradiation (5 Gy) alone gave nearly no increase in pSTAT1 levels at three days post treatment (**Figures 2E, F**). Co-treatment with IR and high concentrations of ATRi (250 nM VE822; 1250 nM AZD6738) markedly increased this response, whereas a smaller increase was obtained with the lower concentrations (50 nM VE822; 250 nM AZD6738) (**Figures 2E, F**). The biggest effect was obtained with the high concentration of VE822 (250 nM), which also caused the highest reduction of cell viability (**Supplementary Figure S1A**). At six days post treatment, IR alone induced the highest pSTAT1 levels, but this induction nevertheless appeared lower than after the aforementioned high-concentration co-treatments at three days (**Figures 2E, F**; **Supplementary Figure S1C**). ATRi thus causes an earlier and more pronounced wave of IFN response, which declines with time. The latter might be related to reduced kinase activities in dying or dead cells. Indeed, the higher concentrations of ATRi rendered measurements unattainable at six days due to too much cell death (data not shown). We also observed increased levels of total STAT1 after the treatments (**Figure 2E**; **Supplementary Figure S1D**), consistent with previous work in other cell lines showing that radiation-induced increase in pSTAT1 is accompanied by increased levels of total STAT1 (18). Of note, a previous study has reported that the cGAS–STING–IFN pathway is defective in U2OS cells due to very low or undetectable expression levels of *STING1* (31). However, we consistently observed an increase in STING level after treatment with IR and ATRi (**Figure 2G**), supporting that this pathway may likely be active in U2OS cells after the treatment.

To verify that pSTAT1 levels represent an activated type I interferon signaling cascade, we measured levels of IFN-β in growth medium supernatants by ELISA three days post treatment. Whereas the unirradiated mock samples and the samples treated with IR or ATRi alone failed to give detectable levels of IFN-β, the combined treatment with IR + 250 nM VE822 – which produced the highest increase in pSTAT1 levels – gave clearly elevated IFN-β concentrations in the medium (**Figure 2H**; **Supplementary Figure S1E**). The ELISA measurements thus confirm that the increased pSTAT1 levels correlated with IFN-β secretion. Altogether, these results indicate that whereas IR alone induces an IFN response at around six days, the combined treatment with IR and ATRi can induce an expedited response at three days post treatment.

### The effect of co-treatment with IR and ATRi on interferon signaling varies between human lung cancer cell lines

**Figure 3:**
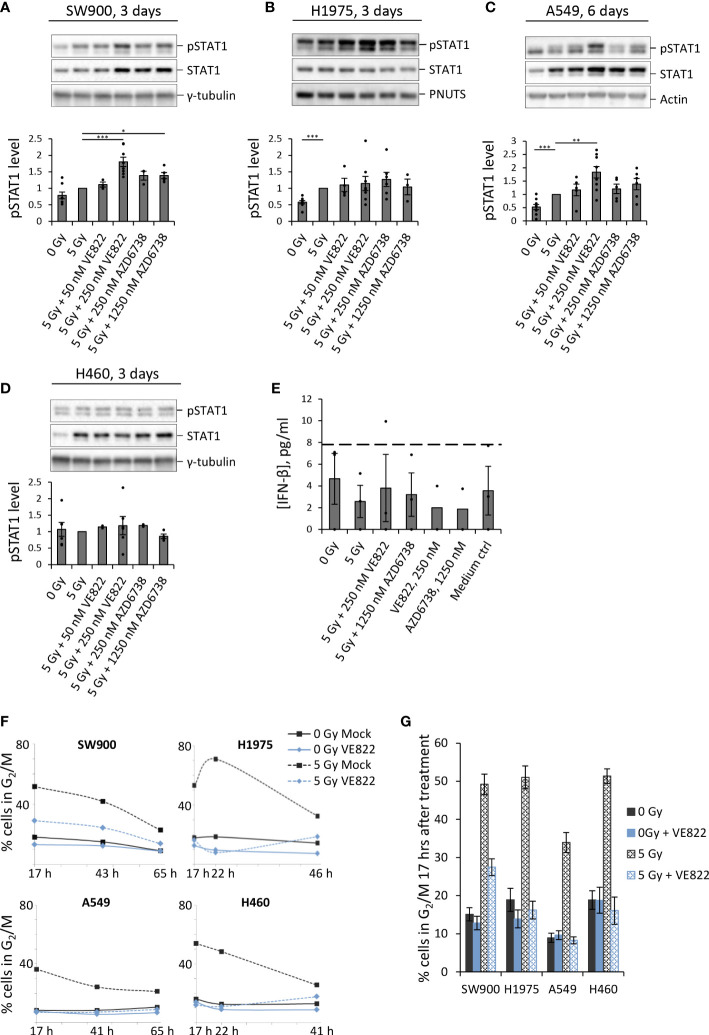
ATRi abrogates radiation-induced G2 checkpoint arrest in four human lung cancer cell lines, and gives increased IFN response in three of these. **(A–D)** Immunoblots for indicated NSCLC cell lines after treatment with IR and ATRi. Bar charts show pSTAT1 levels relative to loading controls and normalized to 5 Gy. (Results for A549 at three days after treatment and at six days for the other cell lines are shown in **Supplementary Figure S2**). γ-tubulin, PNUTS and pan-actin were used as loading controls. **(E)** ELISA of IFN-β in H460 cells treated and analyzed as in **Figure 2H**. (The zero values are from an experiment where all IFN readings were equal to or lower than the lowest value of the standard curve). **(F)** Proportion of cells in G2/M phase after treatment with IR and 250 nM VE822. The corresponding DNA histograms are shown in **Supplementary Figure S3**. **(G)** Bar-plotted quantification of G2/M proportions from three independent experiments performed as in **(F)**, at 17 hours after treatments.

As done for U2OS, we next assayed pSTAT1 levels in the non-small cell lung cancer (NSCLC) cell lines SW900, H1975, A549 and H460. Irradiation alone caused a small increase in pSTAT1 for SW900 and H1975 at three days post treatment, and in A549 at six days post treatment (**Figures 3A–C**). We detected further increased pSTAT1 levels upon co-treatment with IR and ATRi for SW900, H1975 and A549 (**Figures 3A–C**), albeit to a lesser extent than for U2OS. The highest levels of pSTAT1 were observed after treatment with IR + 250 nM VE822 for SW900 and A549 (**Figures 3A, C**), in concordance with the results for U2OS (**Figures 2F, H**). For H1975, the differences between IR and IR + ATRi were not statistically significant, but nevertheless, the pSTAT1 level was increased both after IR alone and in combination with ATRi when compared to the non-irradiated cells (**Figure 3B**). At six days post treatment, all the treatments of H1975 resulted in pSTAT1 levels around or below the mock sample background level (**Supplementary Figure S2C**). SW900, on the other hand, showed a marked radiation-induced increase in pSTAT1 level at six days, but still lower than after IR + ATRi at three days (**Supplementary Figures S2A, B**), resembling the results for U2OS.

Notably, no increase in pSTAT1 levels was observed for H460 after treatment with either IR alone or in combination with ATRi, neither at three nor six days post treatment (**Figure 3D**; **Supplementary Figure S2E**). This was confirmed by ELISA measurements of IFN-β in H460 (**Figure 3E**). H460 thus deviates from the other tested cell lines, all of which showed an increase in pSTAT1 levels after treatment with IR and/or IR + ATRi. To address whether H460 also deviated in terms of G2 checkpoint abrogation, we performed cell cycle analyses after treatment with 5 Gy IR + 250 nM VE822. However, all four lung cancer cell lines showed a clear G2 arrest at 17 hours after irradiation, which was abrogated upon ATR inhibition (**Figures 3F, G**; **Supplementary Figures S3A, B**). Of note is that A549 had less accumulation of cells in G2 phase after irradiation, likely due to a more pronounced G1 checkpoint (**Supplementary Figure S3A**). Thus, A549 may cycle more slowly than the other cell lines after the treatment, which could possibly explain the delayed IFN response in this cell line relative to the others. Together, these results show that ATRi can increase the IFN response after irradiation in three out of the five cell lines tested, and weakly in further one cell line, while the G2 checkpoint was abrogated in all five cell lines.

### Increased pSTAT1 levels after combined treatment with IR and ATRi are dependent on cGAS

**Figure 4:**
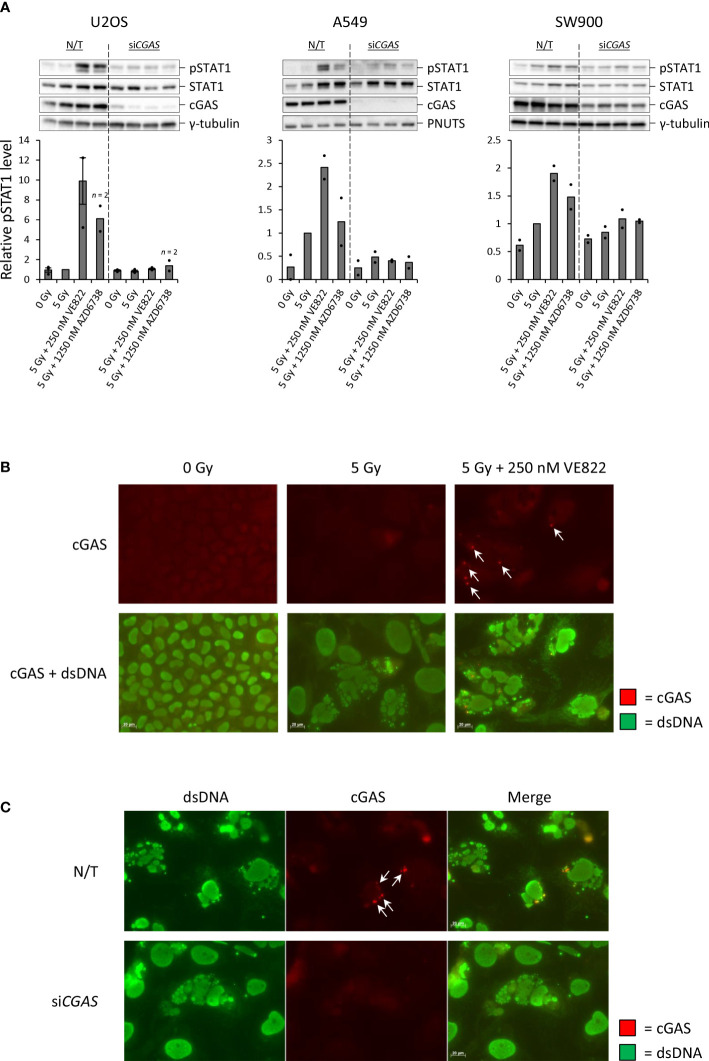
*Increased IFN signaling after IR and ATRi is dependent on the cytosolic DNA sensor cGAS.***(A)** Immunoblots and quantifications of pSTAT1 levels in non-transfected (N/T) and si*CGAS*-transfected cells at three (U2OS, SW900) or six (A549) days after treatment. Bar charts show pSTAT1 levels relative to loading controls (γ-tubulin or PNUTS) and normalized to 5 Gy. **(B)** Micrographs of U2OS cells stained with antibodies against cGAS (red) and dsDNA (green) at three days after treatment. **(C)** Micrographs of U2OS cells transfected with si*CGAS* as in **(A)** and harvested at three days after treatment with 5 Gy and 250 nM VE822. Arrows in Figure B-C indicate cGAS foci localized to micronuclei. Scale bars: 20 µm.

To investigate whether the treatment-induced increases in pSTAT1 levels were dependent on the cytosolic DNA sensor cGAS, we performed siRNA transfection to deplete cGAS in U2OS, A549 and SW900. For all three cell lines, the increase in pSTAT1 level was abolished or heavily diminished upon cGAS depletion (**Figure 4A**). This result substantiates the hypothesis of IFN secretion in response to detection of cytosolic DNA by cGAS after treatment with IR and ATRi. To further elucidate cGAS’ role in the response, we performed immunofluorescence microscopy of U2OS at three days after treatment with IR with and without 250 nM VE822. If cGAS initiates the type I IFN response after detection of *de facto* cytosolic DNA in micronuclei, cGAS should localize to the micronuclear lumen. Indeed, cGAS formed distinct foci localized to micronuclei in U2OS cells after the combined treatment (**Figure 4B**). Transfection with siRNA targeting *CGAS* abolished this effect despite presence of micronuclei (**Figure 4C**). Furthermore, siRNA-mediated depletion of STING also abolished the IFN response after IR and ATRi, highly consistent with activation of the cGAS–STING–IFN pathway in U2OS cells (**Supplementary Figure S4A**). In contrast, transfection with three different non-targeting control siRNAs did not eliminate the IFN response (**Supplementary Figure S4A**). Taken together, these results show that the IFN response is dependent on cGAS–STING, and that there is a link between micronuclear cGAS localization and induction of the type I IFN response.

### Caspase inhibition restores the IFN response in H460 cells and increases the responses in the other cell lines.

**Figure 5:**
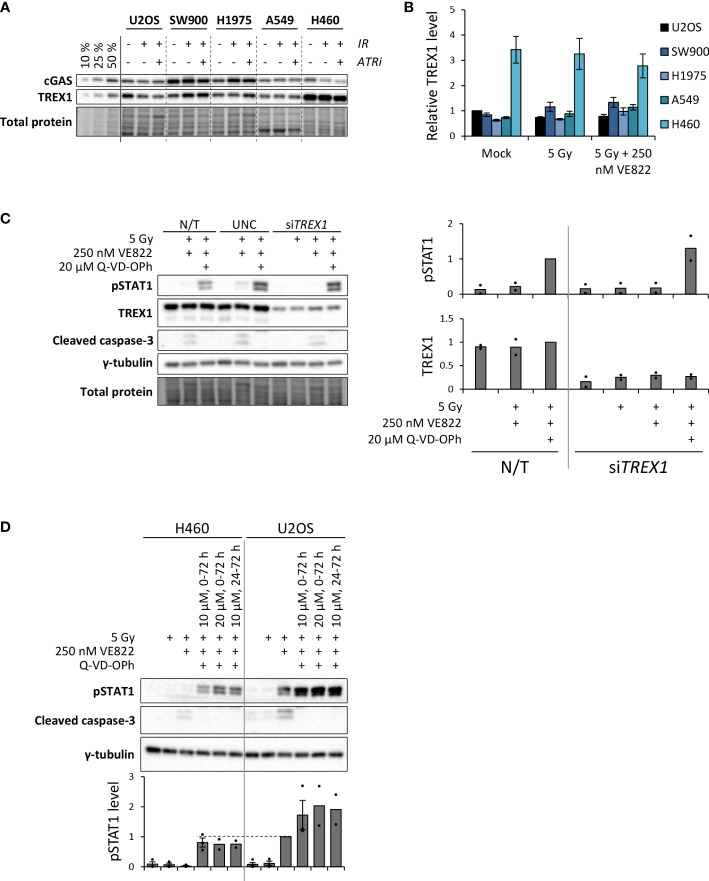
Caspase inhibition restores IFN signaling in the H460 cell line, which initially lacked the IFN response after IR + ATRi. **(A)** Immunoblots showing cGAS and TREX1 levels at three days after treatment. Three leftmost lanes: 10, 25 and 50% loading of the co-treated SW900 sample. ATRi: 250 nM VE822. **(B)** Quantification of immunoblots from three independent experiments as shown in **(A)** for TREX1, relative to total protein levels and normalized to U2OS mock. **(C)** Left: Immunoblots of H460 cells at three days after treatment with IR (5 Gy), ATRi (250 nM VE822) and a pan-caspase inhibitor (20 µM Q-VD-OPh). Cells were transfected with control siRNA (UNC; universal negative control) or siRNA targeting TREX1 at six hours prior to the treatment. Right: Quantification of immunoblots for pSTAT1 and TREX1 from two independent experiments, relative to total protein and normalized to the triple-treated non-transfected (N/T) cells (third lane). **(D)** Immunoblots of H460 cells and U2OS cells at three days after the indicated treatments. The caspase inhibitor was present at 10 µM or 20 µM for 0-72 h or 24-72 h after irradiation, as indicated. The ATR inhibitor (VE822) was present for 0-72 h. Bottom bar chart shows quantification of pSTAT1 levels from three (two for the two latter triple-treatments for both cell lines) independent experiments, relative to γ-tubulin and normalized to the co-treated U2OS sample (5 Gy + 250 nM VE822). Dashed line is included to compare pSTAT1 levels for the triple treated H460 cells with the co-treated U2OS cells.

**Figure 6:**
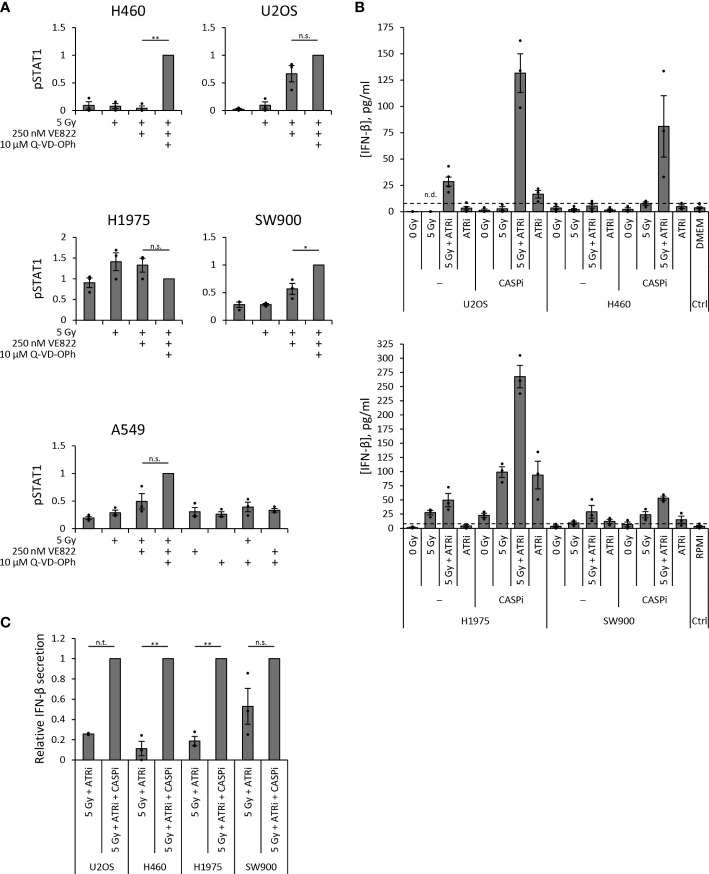
Caspase inhibition increases secreted IFN-β. **(A)** Quantification of pSTAT1 levels from three independent immunoblot experiments in each cell line at three days (H460, U2OS, H1975, SW900) or six days (A549) post treatment. Values are relative to γ-tubulin or total protein and normalized to the triple-treated sample. One sample *t* test was conducted for differences between co-treated (5 Gy + ATRi) and triple-treated (5 Gy + ATRi + CASPi) samples. **(B)** ELISA measurements of secreted IFN-β in 20X upconcentrated growth medium supernatants from samples three days after IR and ATRi. ATRi: 250 nM VE822, CASPi: 10 µM Q-VD-OPh (24–72 h). Top: U2OS and H460 with DMEM medium control; bottom: H1975 and SW900 with RPMI medium control. Results from **Figures 2H** and **3E** are included in the plots for U2OS and H460 without CASPi. **(C)** The IFN-β values in **(B)** for co-treated (5 Gy + ATRi) normalized to the values for triple-treated (5 Gy + ATRi + CASPi) samples. (n.t.: not tested (U2OS, *n* = 2), n.s.: not significant).

As H460 deviated from the other cell lines by the lack of IFN response after treatment, and as TREX1 can degrade the DNA substrate of cGAS, we assessed the protein level of TREX1 in all the cell lines (**Figure 5A**). The level of TREX1 was considerably higher in H460 than in the other cell lines (**Figures 5A, B**), which could imply that TREX1 is responsible for the lack of IFN response in H460. To address this, we depleted TREX1 by siRNA transfection. However, depletion of TREX1 in H460 caused massive cell death and growth arrest, and did not produce any IFN response upon treatment with IR and ATRi (data not shown). We therefore titrated the siRNA concentration to obtain a partial depletion of TREX1 in H460, reaching approximately similar level of TREX1 as in the other cell lines (**Figure 5C**). In this experiment we also included a pan-caspase inhibitor (Q-VD-OPh) to address whether apoptotic cell death might camouflage the effect of TREX1 depletion. Remarkably, the caspase inhibition, but not the TREX1 depletion, resulted in a high pSTAT1 level after treatment with IR and ATRi in H460 (**Figure 5C**). The magnitude of this response after the triple-treatment was comparable to the IFN response in U2OS cells after IR + ATRi (**Figure 5D**). The caspase inhibitor also increased the pSTAT1 levels after IR + ATRi in U2OS, SW900 and A549 cells, but no increase was seen in H1975 cells (**Figure 6A**). Of note is that these differences were not statistically significant for U2OS and A549, but all the experiments anyway showed a similar trend (**Figure 6A**). To further validate these findings, we measured IFN-β by ELISA in H460, U2OS, H1975 and SW900 cells after treatment with ATRi and/or IR in the presence and absence of the caspase inhibitor. The ELISA results confirmed that caspase inhibition restores the IFN response in H460 and increases the responses in U2OS and SW900 cells (**Figures 6B, C**). Intriguingly, caspase inhibition also increased IFN-β secretion in H1975 cells (**Figures 6B, C**), despite the lack of increase in pSTAT1 level (**Figure 6A**). The amount of secreted IFN-β was even higher for H1975 than for the other cell lines. The pSTAT1 response occurring downstream of IFN-β secretion must thus somehow be downregulated in H1975 cells. Treatment-induced cleavage of caspase-3 and PARP1 were detected in H460, U2OS and H1975 cells (**Supplementary Figure S4B**), which also were the three cell lines showing biggest increases in IFN response upon caspase inhibition. Altogether, these results strongly suggest that treatment-induced caspase activation is responsible for the lack of IFN response in H460 cells after IR + ATRi. Furthermore, caspase activation also counteracts the IFN response in the other cell lines.

## Discussion

**Figure 7:**
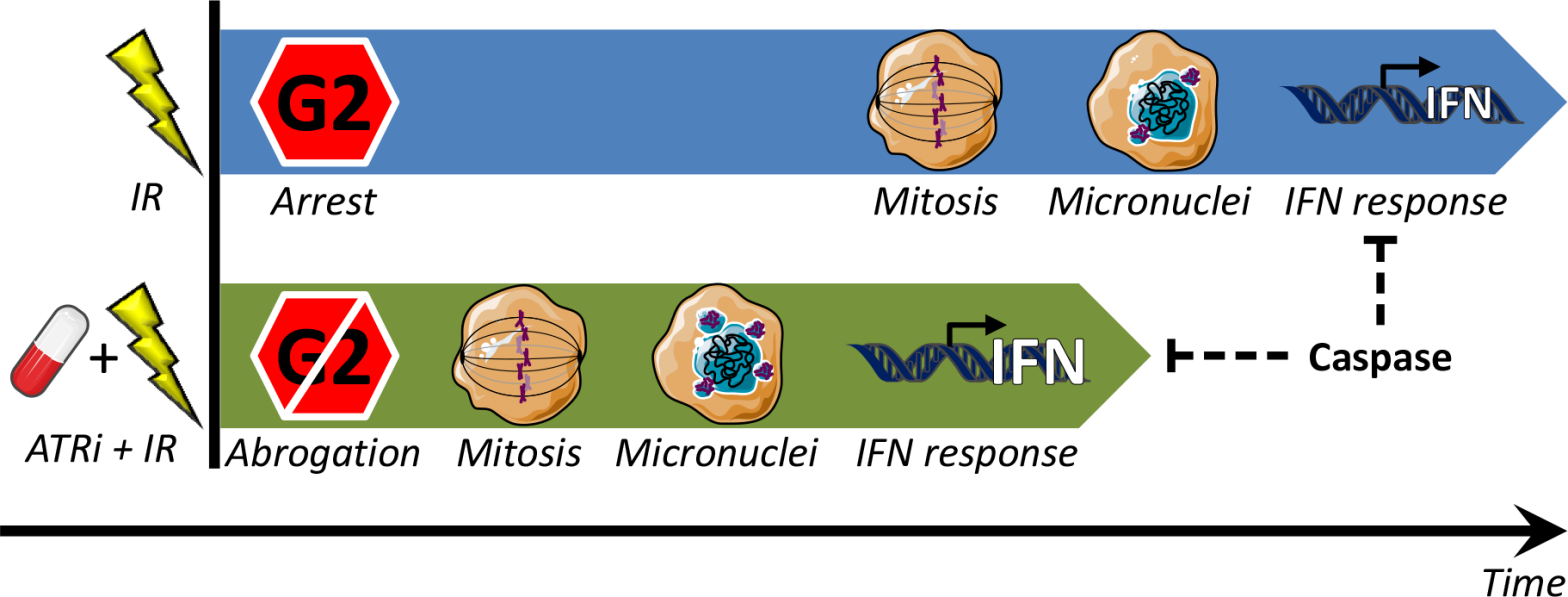
Model for regulation of type I IFN response by G2 checkpoint arrest. Treatment with IR alone (top) can induce a delayed IFN response, occurring after completion of the IR-induced G2 checkpoint arrest. When the G2 arrest is abrogated by ATRi (ATRi + IR; bottom), the IFN response comes earlier, and it is also stronger (because more micronuclei are formed when there is less time for DNA repair prior to mitosis). In both cases IFN is induced due to immune recognition of DNA from ruptured micronuclei, *via* the cGAS–STING–IFN pathway. This pathway can be suppressed by treatment-induced caspase activation.

Combined treatment with ATRi and radiotherapy is a promising strategy under evaluation in clinical trials (10, 11, 32). While the rationale until recently has been ATR’s function in DNA damage repair and cell cycle checkpoints, a new role for ATR is also emerging in the suppression of antitumor immune responses [reviewed in (33–35)]. However, the mechanisms of how ATRi regulate immune effects, and to what extent these are important in human cancers, have been unclear. We show that the ATR inhibitors VE822 and AZD6738 can potentiate radiation-induced, cGAS-dependent type I interferon signaling in several cell lines from human osteosarcoma and NSCLC. On the other hand, IFN signaling was not observed in one of the NSCLC cell lines, H460, despite abrogation of the G2 checkpoint and presence of micronuclei. Remarkably, upon addition of a pan-caspase inhibitor, the IFN response was restored in this cell line after irradiation and ATR inhibition. Moreover, the caspase inhibitor also increased the IFN responses in the other cell lines. Our results are consistent with a model where the ATR inhibitors’ abrogating effect on the G2 checkpoint leads to an IFN response *via* detection of micronuclear DNA by the cytosolic DNA sensor cGAS. The ATR inhibitors thereby accelerate and increase the radiation-induced IFN response. However, treatment-induced caspase activation can suppress this response (**Figure 7**).

Our finding, that caspase inhibition increases interferon signaling, is consistent with previous studies showing caspase-dependent suppression of the cGAS–STING–IFN pathway during DNA virus infection [reviewed in (23)]. A previous study has reported that caspase inhibition also can increase radiation-induced IFN secretion (36). However, to our knowledge, it has not previously been shown that caspase inhibition increases the IFN response after combined treatment with IR and ATRi. We propose that treatment-induced caspase activation counteracts the IFN response mediated by cGAS-detection of DNA from ruptured micronuclei. This finding may partly explain why different cancer cell lines show large variations in the extent of IFN response after irradiation and ATR inhibition [this study and (17)]. In some cell lines, the treatment induces strong caspase activation which suppresses the response. The underlying molecular mechanism of how pan-caspase inhibition increases the IFN response after IR and ATRi remains to be elucidated. Caspases may potentially cleave cGAS or other factors in the cGAS–STING signaling cascade (23, 37). Furthermore, the previous study with radiation-induced IFN suggested that caspase inhibition prevents breakdown of irradiated cells with cytosolic DNA (36). Notably, the IFN response is regulated by multiple factors. In addition to the micronuclei, mitochondrial DNA or endogenous retroviruses can also cause IFN induction after irradiation (38, 39). An important task for the future is therefore to better understand the relative contribution from each of these pathways.

The lack of IFN response in H460, after both irradiation alone and co-treatment with IR and ATRi, coincided with a higher baseline level of TREX1 in this cell line than in the responding ones. We therefore hypothesized that TREX1 might be a regulating factor for the cGAS–IFN signaling pathway in H460. However, neither partial depletion nor full depletion of TREX1 by siRNA transfection did increase IFN signaling in H460. Furthermore, we failed to see an increase in TREX1 levels after treatment with high radiation doses (10-20 Gy). The observed reduction of IFN response after high doses did therefore not correlate with an induction of *TREX1* expression, in contrast to the results of a previous study (25). However, while we assessed TREX1 protein levels, the previous study examined *TREX1* mRNA levels and also applied other cell lines than us, which might explain differences between the results.

In our study, we employed two different concentrations of the ATR inhibitors. While the highest, most toxic concentrations of the inhibitors (250 nM VE822; 1250 nM AZD6738) abrogated the G2 checkpoint and induced IFN signaling, the lower concentrations (50 nM VE822; 250 nM AZD6738), which were less toxic, only moderately abrogated the checkpoint and showed minor increases in IFN signaling. Of note is that the higher inhibitor concentrations are toxic even without irradiation, and the concentrations typically used for radiosensitization of cancer cell lines are closer to the lower concentrations in our study. Radiosensitizing effects have for instance been reported with 25-50 nM VE822 in U2OS and A549 cells (40) and with 100-300 nM AZD6738 in A549 and H460 cells (41), as measured by clonogenic survival. Interestingly, in order to cause pronounced increases in IFN signaling, the cells required higher concentrations of the inhibitors than what is needed for a mere radiosensitizing effect. The effects of ATRi in IFN signaling nevertheless required co-treatment with radiation, as treatment with the inhibitors in the absence of irradiation caused no or only small increases in IFN response (**Figures 2H**; **3E**; **6A, B**).

The reduction in IFN response after high IR doses (10-20 Gy) correlated with a prolonged G2 checkpoint arrest. This correlation is in line with previous reports showing reduced IFN signaling and a longer G2 checkpoint arrest after irradiation of DNA repair-deficient cells, as compared to repair-proficient cells (16, 18). In repair-deficient cells, the higher level of unrepaired DNA damage with low radiation doses will cause a longer G2 checkpoint arrest, analogous to the prolonged checkpoint arrest seen in repair-proficient cells with high radiation doses. Our results thus strongly support the notion that radiation-induced cell cycle arrest functions to suppress the type I IFN response (16). Previously, a phenomenon of checkpoint adaptation and G2 checkpoint imperfectness, allowing cells to escape checkpoint arrest even with remaining DNA breaks, has been described (42, 43). Interestingly, the link between micronuclei and induction of IFN signaling suggests an important functional role of checkpoint adaptation in stimulating antitumor immune responses.

In conclusion, the combined treatment of irradiation and ATR inhibition can potentiate radiation-induced type I IFN responses, and thus be a candidate immunostimulatory radiotherapeutic strategy. The clinically relevant immune effect of such co-treatment will likely depend on the type of cancer, the heterogeneity of the tumors and possibly also treatment-induced caspase activation. Adding caspase inhibitors could potentially also be a future strategy to increase antitumor immune effects, although it is far from clear how they will affect both normal tissue and other antitumor responses. Further *in vivo* investigation will unveil the fuller potential of these combined treatments, which may also be further combined with immune checkpoint inhibition.

## Materials and methods

### Cell culture, irradiation and inhibitor treatment

Human H460 and A549 NSCLC and U2OS osteosarcoma cells were grown in DMEM with GlutaMAX-I, and SW900 and H1975 NSCLC cells in RPMI 1640 medium with GlutaMAX-I (both media from Gibco by Life Technologies), at 37°C with humidified 5% CO_2_ atmosphere. The media were supplemented with 10% fetal bovine serum (Biowest) and 1% penicillin–streptomycin solution (50 IU/ml) (Gibco). Cells were tested for *Mycoplasma* infection, and their identity was confirmed by short tandem repeat analysis. ATR inhibitors VE822 (berzosertib/VX970, Selleckchem) and AZD6738 (ceralasertib, Selleckchem) were added 10–30 minutes before irradiation (160 kV X-rays, 1 Gy/min, Faxitron CP-160).

### Cell cycle analysis

Cells were fixated with 70% ethanol, stained with Hoechst 33258 (Sigma-Aldrich) and analyzed with a LSR II flow cytometer (BD Biosciences) coupled to the BD FACSDiva v8 software. DNA histograms were analyzed in FlowJo v10. Cell cycle analysis was conducted by the Watson algorithm.

### Immunoblotting

Cells were lysed in whole-cell lysis buffer (20 mM NaCl, 2 mM MgCl_2_, 50 mM Tris-HCl pH 7.5, 0.5% Triton X-100) with protease and phosphatase inhibitor cocktails (cOmplete mini (EDTA-free) and PhosSTOP EASYpack, Roche) and benzonase (100 IU/ml; Merck/Sigma-Aldrich). Protein concentration was measured by Micro BCA Protein Assay kit (ThermoFisher Scientific), and adjusted. Lane Marker Reducing Sample Buffer (Pierce) was added and the samples were boiled for 10 minutes at 95°C. SDS-polyacrylamide 4-15% gradient gels (Bio-Rad) were used for electrophoresis and nitrocellulose membranes (Bio-Rad) for blotting. The resulting membrane was blocked in 5% non-fat skimmed-milk powder in PBS with 0.1% Tween (PBST) at room temperature for a minimum of 30 minutes. Membranes were stained with primary antibodies at 4°C over-night and secondary antibodies at room temperature for 30-45 minutes (antibodies were diluted in the aforementioned blocking solution), before addition of enhanced chemiluminescence solution (ThermoFisher Scientific). Washing of membranes after transfer and antibody incubations was done in room-tempered PBST. Images were processed and quantifications were performed in Image Lab 4.1 (Bio-Rad). Range of detection was verified by excluding saturated signals and by including a dilution series of one of the samples (see **Figure 5A**). The resulting standard curve allowed for accurate quantification. Antibodies are listed in **Supplementary Table 1**.

### Immunofluorescence microscopy

Cells were cultured on glass coverslips and fixated with 10% formalin solution (Sigma-Aldrich) for 10 minutes at room temperature. Cells were permeabilized with 0.2% Triton X-100 (Sigma-Aldrich) in PBS, and stained with primary antibodies for 1 hour followed by secondary antibodies for 30 minutes. For blocking, the antibodies were diluted in room-tempered DMEM with 10% FBS upon staining of coverslips. The coverslips were washed three times in PBS after fixation, permeabilization and antibody incubations. Coverslips were mounted with mowiol solution (Sigma-Aldrich). Antibodies are listed in **Supplementary Table 2**.

### siRNA transfection for gene knockdown

For cGAS depletion, cells were transfected with 20 nM si*CGAS* (M-015607-01-0005, SMARTpool, Dharmacon). For STING depletion, cells were transfected with 10 nM si*STING1* (si*TMEM173*, ID 128591, Ambion). For partial TREX1 depletion, cells were transfected with 5 nM si*TREX1* (ID s535182, Ambion). All transfections were performed with Lipofectamine RNAiMax (Invitrogen), at six hours before treatment. For siRNA sequences, consult **Supplementary Table 3**.

### Enzyme-linked immunosorbent assay (ELISA) of interferon-β

Growth medium supernatants were centrifuged to exclude floating cells. Resulting supernatants were 20X up-concentrated by centrifuge filtering through 10 kDa cut-off columns (Amicon Ultracel-10, Merck). ELISA (Human IFN-beta DuoSET ELISA, R&D Systems) was conducted according to supplier’s protocol. Optical density was measured at 450 nm with pathlength correction at 540 nm in a microplate spectrophotometer (PowerWave XS2, BioTek) coupled to the Gen5 software v2.09.1. IFN-β standards were included in all experiments, and a best-fitting 2^nd^ degree polynomial function was used for calculation of measured IFN-β in the samples.

### Statistics

Error bars represent standard error of the mean (SEM; *n* ≥ 3). Dots in bar charts indicate individual experiments. *p* values (one-sample Student’s *t* test for pairs involving normalization value (*i.e.* 5 Gy for most plots); two-tailed, paired-samples Student’s *t* test for the remaining pairs) were calculated with IBM SPSS Statistics v28, with significance level set to 0.05. *p ≤ 0.05, **p ≤ 0.01, ***p ≤ 0.001.

## Data availability statement

The raw data supporting the conclusions of this article will be made available upon request to the corresponding author.

## Author contributions

Conceptualization: RGS, SH, AEM. Experiments: AEM, SH, GER. Supporting experiments: IØ. Data analysis: AEM, SH, IØ, RGS. Figures: AEM, SH. Supervision: RGS, AC. Critical review of work: all authors. Writing—original draft preparation: AEM, RGS. Writing—editing: all authors. Funding acquisition: RGS. All authors contributed to the article and approved the submitted version.

## Funding

This work was funded by grants from the South-Eastern Norway Regional Health Authorities (2018010) and the Norwegian Cancer Society (198018).

## Acknowledgments

We thank the flow cytometry core facility at the Norwegian Radium Hospital, Oslo University Hospital for training and useful support. We also thank Karoline Kongsrud for technical assistance regarding **Figure S4B**.

## Conflict of interest

The authors declare that the research was conducted in the absence of any commercial or financial relationships that could be construed as a potential conflict of interest.

## Publisher’s note

All claims expressed in this article are solely those of the authors and do not necessarily represent those of their affiliated organizations, or those of the publisher, the editors and the reviewers. Any product that may be evaluated in this article, or claim that may be made by its manufacturer, is not guaranteed or endorsed by the publisher.

## References

[1] Abuodeh Y Venkat P Kim S. Systematic review of case reports on the abscopal effect. Curr problems cancer (2016) 40(1):25–37. doi: 10.1016/j.currproblcancer.2015.10.001 26582738

[2] Rodríguez-Ruiz ME Vanpouille-Box C Melero I Formenti SC Demaria S. Immunological mechanisms responsible for radiation-induced abscopal effect. Trends Immunol (2018) 39(8):644–55. doi: 10.1016/j.it.2018.06.001 PMC6326574 30001871

[3] Sato H Niimi A Yasuhara T Permata TBM Hagiwara Y Isono M. DNA Double-strand break repair pathway regulates PD-L1 expression in cancer cells. Nat Commun (2017) 8(1):1751. doi: 10.1038/s41467-017-01883-9 29170499 PMC5701012

[4] Ko EC Formenti SC. Radiotherapy and checkpoint inhibitors: a winning new combination? Ther Adv Med Oncol (2018) 10:1758835918768240. doi: 10.1177/1758835918768240 29662550 PMC5898659

[5] Formenti SC Rudqvist NP Golden E Cooper B Wennerberg E Lhuillier C. Radiotherapy induces responses of lung cancer to CTLA-4 blockade. Nat Med (2018) 24(12):1845–51. doi: 10.1038/s41591-018-0232-2 PMC6286242 30397353

[6] Sato H Demaria S Ohno T. The role of radiotherapy in the age of immunotherapy. Japanese J Clin Oncol (2021) 51(4):513–22. doi: 10.1093/jjco/hyaa268 PMC8012351 33561212

[7] Buisson R Niraj J Rodrigue A Ho CK Kreuzer J Foo TK. Coupling of homologous recombination and the checkpoint by ATR. Mol Cell (2017) 65(2):336–46. doi: 10.1016/j.molcel.2016.12.007 PMC5496772 28089683

[8] Iliakis G Wang Y Guan J Wang H. DNA Damage checkpoint control in cells exposed to ionizing radiation. Oncogene (2003) 22(37):5834–47. doi: 10.1038/sj.onc.1206682 12947390

[9] Castedo M Perfettini JL Roumier T Andreau K Medema R Kroemer G. Cell death by mitotic catastrophe: a molecular definition. Oncogene (2004) 23(16):2825–37. doi: 10.1038/sj.onc.1207528 15077146

[10] Dillon MT Boylan Z Smith D Guevara J Mohammed K Peckitt C. PATRIOT: A phase I study to assess the tolerability, safety and biological effects of a specific ataxia telangiectasia and Rad3-related (ATR) inhibitor (AZD6738) as a single agent and in combination with palliative radiation therapy in patients with solid tumours. Clin Trans Radiat Oncol (2018) 12:16–20. doi: 10.1016/j.ctro.2018.06.001 PMC6068075 30073210

[11] Barnieh FM Loadman PM Falconer RA. Progress towards a clinically-successful ATR inhibitor for cancer therapy. Curr Res Pharmacol Drug Discovery (2021) 2:100017. doi: 10.1016/j.crphar.2021.100017 PMC8663972 34909652

[12] Dillon MT Bergerhoff KF Pedersen M Whittock H Crespo-Rodriguez E Patin EC. ATR inhibition potentiates the radiation-induced inflammatory tumor microenvironment. Clin Cancer Res an Off J Am Assoc Cancer Res (2019) 25(11):3392–403. doi: 10.1158/1078-0432.CCR-18-1821 PMC6551222 30770349

[13] Sheng H Huang Y Xiao Y Zhu Z Shen M Zhou P. ATR inhibitor AZD6738 enhances the antitumor activity of radiotherapy and immune checkpoint inhibitors by potentiating the tumor immune microenvironment in hepatocellular carcinoma. J immunotherapy Cancer (2020) 8(1):e000340. doi: 10.1136/jitc-2019-000340 PMC7254123 32461345

[14] Vendetti FP Karukonda P Clump DA Teo T Lalonde R Nugent K. ATR kinase inhibitor AZD6738 potentiates CD8+ T cell-dependent antitumor activity following radiation. J Clin Invest (2018) 128(9):3926–40. doi: 10.1172/JCI96519 PMC6118586 29952768

[15] Sun LL Yang RY Li CW Chen MK Shao B Hsu JM. Inhibition of ATR downregulates PD-L1 and sensitizes tumor cells to T cell-mediated killing. Am J Cancer Res (2018) 8(7):1307–16. PMC6079156 30094103

[16] Chen J Harding SM Natesan R Tian L Benci JL Li W. Cell cycle checkpoints cooperate to suppress DNA- and RNA-associated molecular pattern recognition and anti-tumor immune responses. Cell Rep (2020) 32(9):108080. doi: 10.1016/j.celrep.2020.108080 32877684 PMC7530826

[17] Feng X Tubbs A Zhang C Tang M Sridharan S Wang C. ATR inhibition potentiates ionizing radiation-induced interferon response *via* cytosolic nucleic acid-sensing pathways. EMBO J (2020) 39(14):e104036. doi: 10.15252/embj.2019104036 32484965 PMC7361286

[18] Harding SM Benci JL Irianto J Discher DE Minn AJ Greenberg RA. Mitotic progression following DNA damage enables pattern recognition within micronuclei. Nature (2017) 548(7668):466–70. doi: 10.1038/nature23470 PMC5857357 28759889

[19] Mackenzie KJ Carroll P Martin CA Murina O Fluteau A Simpson DJ. cGAS surveillance of micronuclei links genome instability to innate immunity. Nature (2017) 548(7668):461–5. doi: 10.1038/nature23449 PMC5870830 28738408

[20] Sun L Wu J Du F Chen X Chen ZJ. Cyclic GMP-AMP synthase is a cytosolic DNA sensor that activates the type I interferon pathway. Sci (New York NY) (2013) 339(6121):786–91. doi: 10.1126/science.1232458 PMC3863629 23258413

[21] Mohr L Toufektchan E von Morgen P Chu K Kapoor A Maciejowski J. ER-directed TREX1 limits cGAS activation at micronuclei. Mol Cell (2021) 81(4):724–38.e9. doi: 10.1016/j.molcel.2020.12.037 33476576 PMC7897315

[22] Stetson DB Ko JS Heidmann T Medzhitov R. Trex1 prevents cell-intrinsic initiation of autoimmunity. Cell (2008) 134(4):587–98. doi: 10.1016/j.cell.2008.06.032 PMC2626626 18724932

[23] Fang Y Peng K. Regulation of innate immune responses by cell death-associated caspases during virus infection. FEBS J (2022) 289(14):4098–111. doi: 10.1111/febs.16051 34089572

[24] Schindler C Shuai K Prezioso VR Darnell JE Jr. Interferon-dependent tyrosine phosphorylation of a latent cytoplasmic transcription factor. Sci (New York NY) (1992) 257(5071):809–13. doi: 10.1126/science.1496401 1496401

[25] Vanpouille-Box C Alard A Aryankalayil MJ Sarfraz Y Diamond JM Schneider RJ. DNA Exonuclease Trex1 regulates radiotherapy-induced tumour immunogenicity. Nat Commun (2017) 8:15618. doi: 10.1038/ncomms15618 28598415 PMC5472757

[26] Bunz F Dutriaux A Lengauer C Waldman T Zhou S Brown JP. Requirement for p53 and p21 to sustain G2 arrest after DNA damage. Sci (New York NY) (1998) 282(5393):1497–501. doi: 10.1126/science.282.5393.1497 9822382

[27] Menzel T Nähse-Kumpf V Kousholt AN Klein DK Lund-Andersen C Lees M. A genetic screen identifies BRCA2 and PALB2 as key regulators of G2 checkpoint maintenance. EMBO Rep (2011) 12(7):705–12. doi: 10.1038/embor.2011.99 PMC3128973 21637299

[28] Mladenov E Fan X Dueva R Soni A Iliakis G. Radiation-dose-dependent functional synergisms between ATM, ATR and DNA-PKcs in checkpoint control and resection in G(2)-phase. Sci Rep (2019) 9(1):8255. doi: 10.1038/s41598-019-44771-6 31164689 PMC6547644

[29] Shibata A Barton O Noon AT Dahm K Deckbar D Goodarzi AA. Role of ATM and the damage response mediator proteins 53BP1 and MDC1 in the maintenance of G(2)/M checkpoint arrest. Mol Cell Biol (2010) 30(13):3371–83. doi: 10.1128/MCB.01644-09 PMC2897583 20421415

[30] Simhadri S Vincelli G Huo Y Misenko S Foo TK Ahlskog J. PALB2 connects BRCA1 and BRCA2 in the G2/M checkpoint response. Oncogene (2019) 38(10):1585–96. doi: 10.1038/s41388-018-0535-2 PMC6408219 30337689

[31] Deschamps T Kalamvoki M. Impaired STING pathway in human osteosarcoma U2OS cells contributes to the growth of ICP0-null mutant herpes simplex virus. J Virol (2017) 91(9):e00006-17. doi: 10.1128/JVI.00006-17 28179534 PMC5391473

[32] Rundle S Bradbury A Drew Y Curtin NJ. Targeting the ATR-CHK1 axis in cancer therapy. Cancers (2017) 9(5):41. doi: 10.3390/cancers9050041 PMC5447951 28448462

[33] Hauge S Eek Mariampillai A Rødland GE Bay LTE Landsverk HB Syljuåsen RG. Expanding roles of cell cycle checkpoint inhibitors in radiation oncology. Int J Radiat Biol (2021), 1–10. doi: 10.1080/09553002.2021.1913529 33877959

[34] Sun W Zhang Q Wang R Li Y Sun Y Yang L. Targeting DNA damage repair for immune checkpoint inhibition: Mechanisms and potential clinical applications. Front Oncol (2021) 11:648687. doi: 10.3389/fonc.2021.648687 34026622 PMC8137908

[35] McLaughlin M Patin EC Pedersen M Wilkins A Dillon MT Melcher AA. Inflammatory microenvironment remodelling by tumour cells after radiotherapy. Nat Rev Cancer (2020) 20(4):203–17. doi: 10.1038/s41568-020-0246-1 32161398

[36] Rodriguez-Ruiz ME Buqué A Hensler M Chen J Bloy N Petroni G. Apoptotic caspases inhibit abscopal responses to radiation and identify a new prognostic biomarker for breast cancer patients. Oncoimmunology (2019) 8(11):e1655964. doi: 10.1080/2162402X.2019.1655964 31646105 PMC6791460

[37] Xiong Y Tang YD Zheng C. The crosstalk between the caspase family and the cGAS−STING signaling pathway. J Mol Cell Biol (2021) 13(10):739–47. doi: 10.1093/jmcb/mjab071 PMC8718194 34718659

[38] Pan D Bao X Hu M Jiao M Li F Li CY. SETDB1 restrains endogenous retrovirus expression and antitumor immunity during radiotherapy. Cancer Res (2022) 82(15):2748–60. doi: 10.1158/0008-5472.CAN-21-3523 PMC9357127 35648422

[39] Tigano M Vargas DC Tremblay-Belzile S Fu Y Sfeir A. Nuclear sensing of breaks in mitochondrial DNA enhances immune surveillance. Nature (2021) 591(7850):477–81. doi: 10.1038/s41586-021-03269-w 33627873

[40] Rødland GE Hauge S Hasvold G Bay LTE Raabe TTH Joel M. Differential effects of combined ATR/WEE1 inhibition in cancer cells. Cancers (2021) 13(15):3790. doi: 10.3390/cancers13153790 34359691 PMC8345075

[41] Gill SJ Wijnhoven PWG Fok JHL Lloyd RL Cairns J Armenia J. Radiopotentiation profiling of multiple inhibitors of the DNA damage response for early clinical development. Mol Cancer Ther (2021) 20(9):1614–26. doi: 10.1158/1535-7163.MCT-20-0502 PMC8650722 34158341

[42] Deckbar D Birraux J Krempler A Tchouandong L Beucher A Walker S. Chromosome breakage after G2 checkpoint release. J Cell Biol (2007) 176(6):749–55. doi: 10.1083/jcb.200612047 PMC2064048 17353355

[43] Syljuåsen RG Jensen S Bartek J Lukas J. Adaptation to the ionizing radiation-induced G2 checkpoint occurs in human cells and depends on checkpoint kinase 1 and polo-like kinase 1 kinases. Cancer Res (2006) 66(21):10253–7. doi: 10.1158/0008-5472.CAN-06-2144 17079442

