# Supplementary material for: Caspase activation counteracts interferon signaling after G2 checkpoint abrogation by ATR inhibition in irradiated human cancer cells
Source: Front Oncol. 2022 Oct 28;12:981332. doi: 10.3389/fonc.2022.981332 (PMC9650454; doi:10.3389/fonc.2022.981332)
Supplement: Supplementary file 1 [file DataSheet_1.pdf]

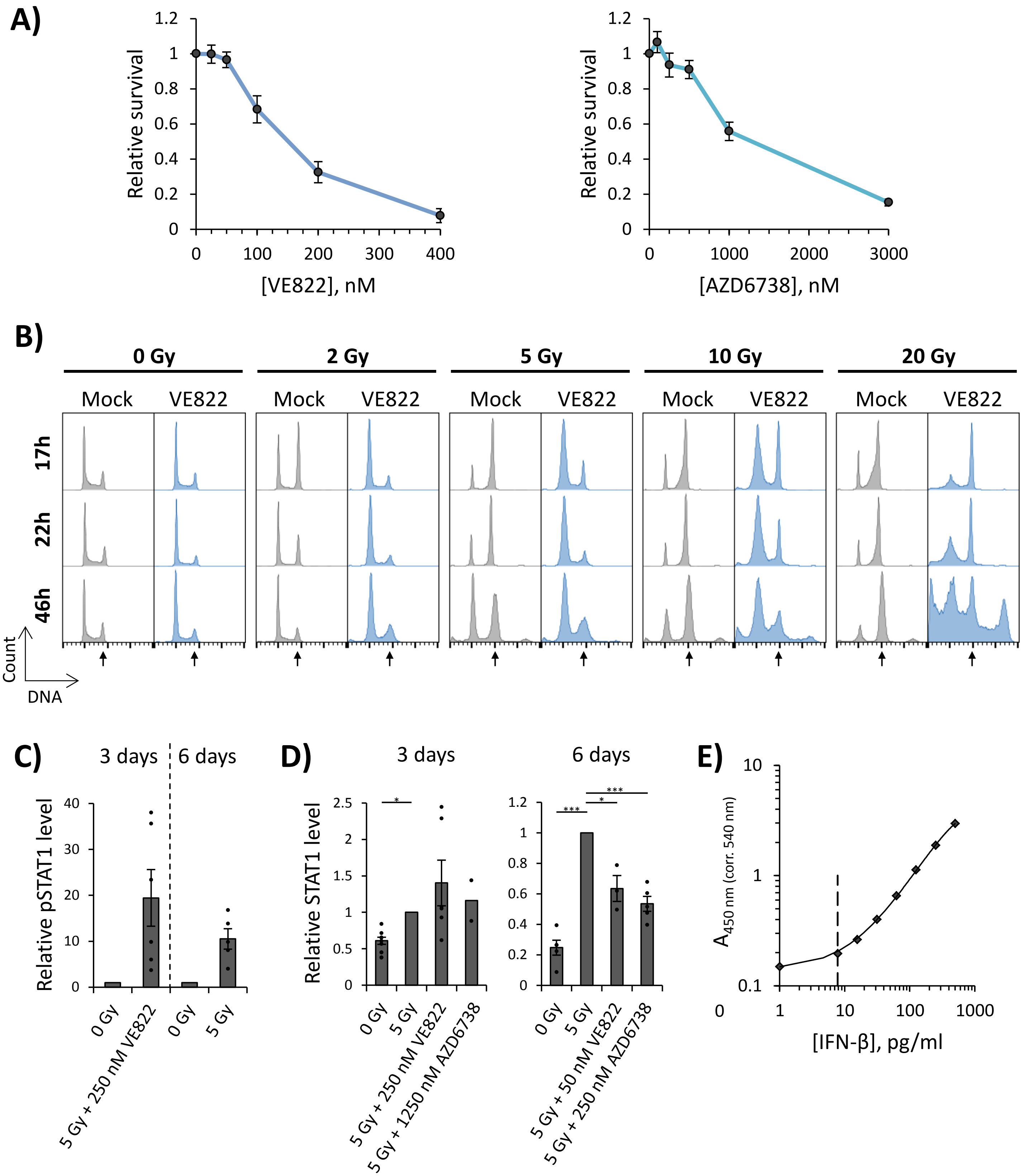

Supplement figure S1. (A) U2OS survival after treatment with various concentrations of the ATR inhibitors VE822 (left) and AZD6738 (right) for 24 hours, assayed by *CellTiter-Glo* viability assay at 3-4 days after drug removal. Cell viability relative to untreated cells is shown. Error bars: SEM (n = 4). **(B)** DNA histograms showing cell cycle distribution in U2OS cells after mock treatment and 2, 5, 10 and 20 Gy irradiation with or without ATR inhibitor VE822 (250 nM) at 17, 22 and 46 hours after treatment. Arrows indicate the G2/M phase peaks. **(C)** Compared pSTAT1 response between 5 Gy + 250 nM VE822 at 3 days and 5 Gy at 6 days post treatment for U2OS, normalized to mock. Error bars: SEM. **(D)** Quantification of total STAT1 levels relative to loading controls for experiments as in figure 2E. Values were normalized to the values for the 5 Gy treatments. Dots indicate individual experiments. Error bars: SEM ($n \geq 3$, except for 5 Gy + 1250 nM AZD6738 at three days, for which n = 2). $*p \leq 0.05$, $**p \leq 0.01$, $***p \leq 0.001$. **(E)** Representative standard curve for IFN-β in the ELISAs in figure 2H, 3E and 6B.

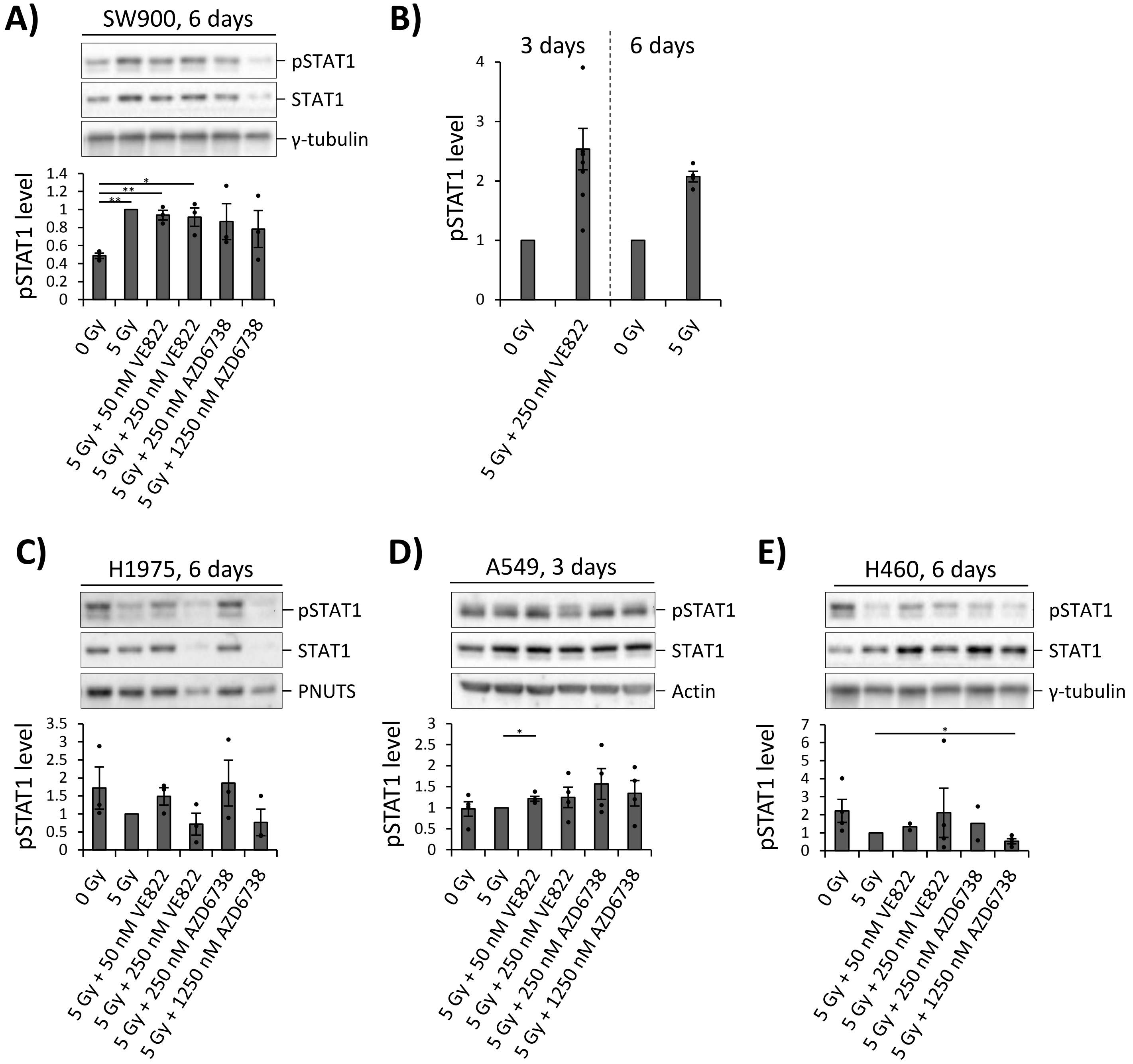

Supplement figure S2. (A, C-D) Immunoblots and quantifications as in figure 3A, at six days (SW900, H1975, H460) or three days (A549) after treatment. γ-tubulin, PNUTS, pan-actin and γ-tubulin were used as loading controls, respectively. Results were normalized to the value for the 5 Gy treatment. Dots indicate individual experiments. Error bars: SEM ($n \geq 3$, except for 5 Gy + 50 nM VE822 and 5 Gy + 250 nM AZD6738 for H460, for which $n = 2$). *$p \leq 0.05$, **$p \leq 0.01$. **(B)** Compared pSTAT1 response between 5 Gy + 250 nM VE822 at 3 days and 5 Gy at 6 days post treatment for SW900, normalized to mock. Error bars: SEM.

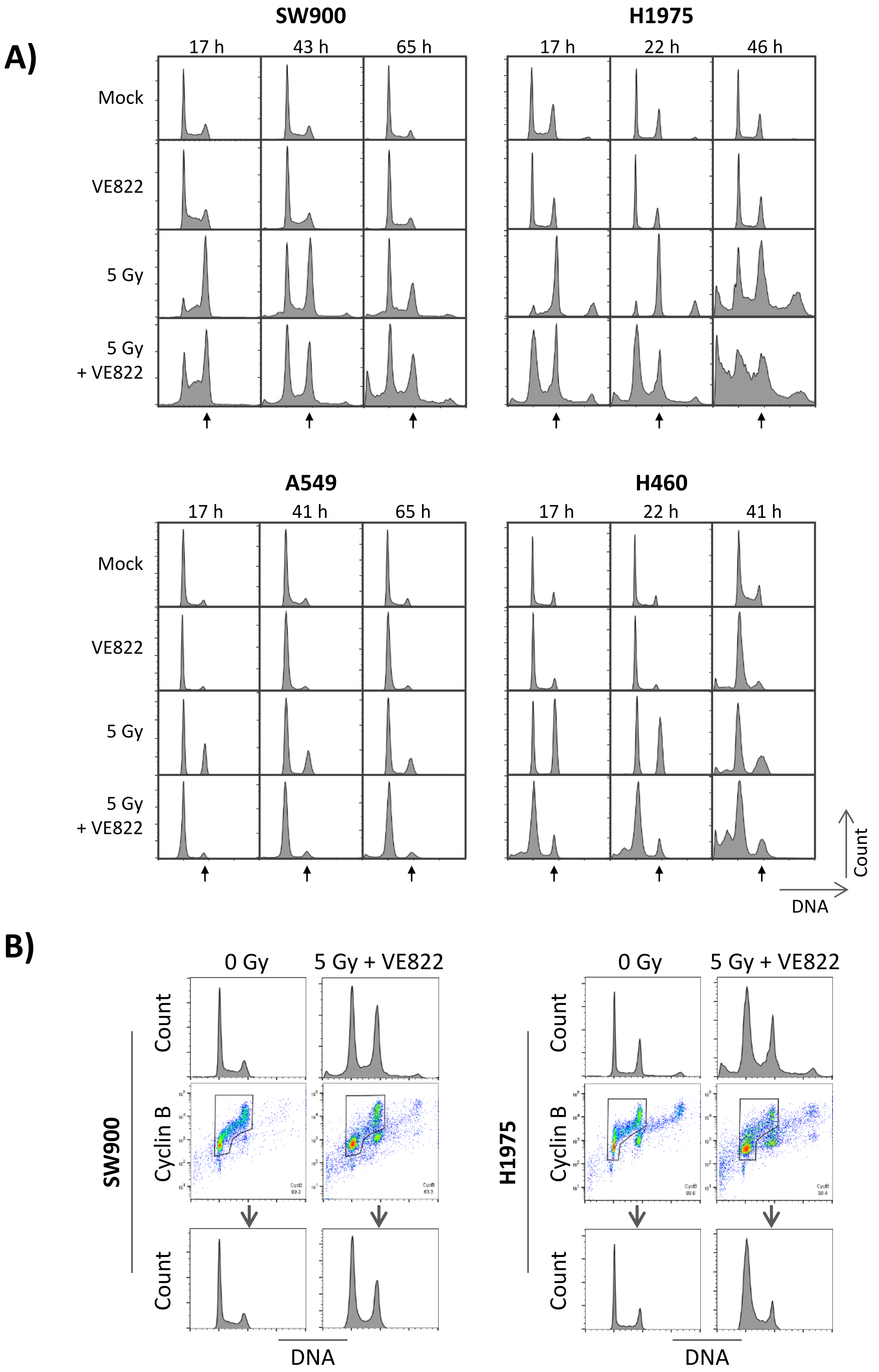

Supplement figure S3. (A) DNA histograms used for the cell cycle analysis in figure 3F. SW900, H1975, A549 and H460 cells were analyzed at the indicated time points after treatment with or without ATR inhibitor VE822 and 5 Gy irradiation. Arrows indicate the G2/M phase peaks. **(B)** Gating strategy employed to the cell cycle analysis of H1975 and SW900 in order to exclude endoreplicating cell fractions. Staining with antibodies against cyclin B was used to identify true G2 phase cells, thus excluding endoreplicating cells with similar DNA content.

A)

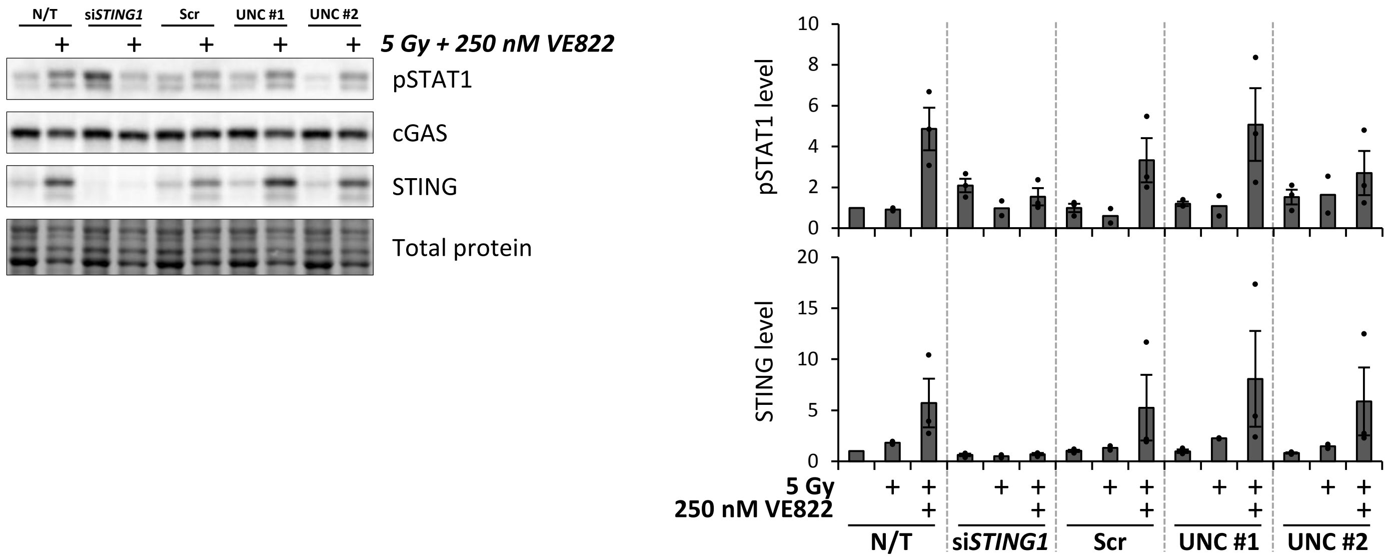

B)

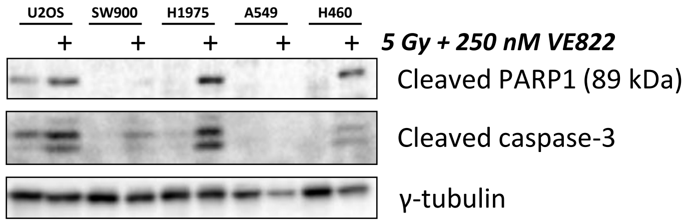

Supplement figure S4. (A) Left: Immunoblots of U2OS cells at three days after treatment with IR + ATRi (5 Gy + 250 nM VE822). Cells were transfected with three different control siRNAs (UNC#1, UNC #2, Scr) or siRNA targeting STING at six hours prior to the treatment. Right: Quantification of immunoblots for pSTAT1 and STING from three independent experiments, relative to total protein and normalized to the non-treated sample (N/T, first lane). **(B)** Representative blots showing the degree of apoptotic response in U2OS, SW900, H1975, A549 and H460 cells with or without treatment with 5 Gy + 250 nM VE822, as measured by cleavage of PARP1 and caspase-3. γ-tubulin is used as loading control.

Supplementary methods:

***CellTiter-Glo* viability assay:**

U2OS cells were seeded onto a 96 well plate and grown over-night, before treatment with ATRi VE822 (25-400 nM) and AZD6738 (100-3000 nM). The inhibitors were washed off after 24 hours, and the cells were supplied with fresh medium. Cell viability was assayed 4-5 days after treatment, by use of the *CellTiter-Glo* Viability Assay (Promega), according to the supplier's protocol. Luminescence was measured spectrophotometrically in a Tecan Spark 10M microplate reader coupled to the software Spark Magellan v1.2 (integration time: 1 s).

Supplementary table 1:

Antibodies used for immunoblotting

	Target (cat.no.)	*Supplier*	*Dilution*
Primary antibodies:	pSTAT1 (#9167)	Cell Signaling Technologies	1:1000
	STAT1 (sc-464)	Santa Cruz Biotechnology	1:200
	cGAS (#15102)	Cell Signaling Technologies	1:1000
	STING (19851-1-AP)	Proteintech	1:1000
	TREX1 (#15107)	Cell Signaling Technologies	1:1000
	Cleaved caspase-3 (#9664)	Cell Signaling Technologies	1:1000
	PARP1 (sc-8007)	Santa Cruz Biotechnology	1:200
	γ-tubulin (T6557)	Sigma-Aldrich	1:2000
	Actin (A-5060)	Sigma-Aldrich	1:2000
	PNUTS (#611060)	BD Biosciences	1:1000
Secondary antibodies:	Horseradish peroxidase conjugated goat anti-rabbit (#111-035-144)	Jackson ImmunoResearch	1:10 000
	Horseradish peroxidase conjugated donkey anti-mouse (#715-035-150)	Jackson ImmunoResearch	1:10 000

Supplementary table 2:

Antibodies used for immunofluorescence microscopy

	Target (cat.no.)	*Supplier*	*Dilution*
Primary antibodies:	cGAS (#15102)	Cell Signaling Technologies	1:200
	dsDNA (ab27156)	Abcam	1:1000
Secondary antibodies:	Alexa Fluor 488 donkey anti-mouse	Molecular Probes by Life Technologies	1:1000
	Alexa Fluor 568 donkey anti-rabbit	Molecular Probes by Life Technologies	1:1000

Supplementary table 3:

siRNA sequences

	Sequence:
**si*CGAS***	5’-GAAGAAACAUGGCGGCUAU-3’<br>5’-GAAGAGAAAUGUUGCAGGA-3’<br>5’-GUAAGGAAUUUCUGACAAA-3’<br>5’-CAACACUCGUGCAUAUUAC-3’
**si*STING1***	5’-GGAUCGGGUUUACAGCAACtt-3’
**si*TREX1***	5’-GACCAUCUGCUGUCACAACtt-3’

Supplementary table 4:

Antibodies used for flow cytometry

	Target (cat.no.)	***Supplier***	***Dilution***
Primary antibody:	Cyclin B (sc-245)	Santa Cruz Biotechnology	1:500
Secondary antibody:	Alexa Fluor 488 donkey anti-mouse	Molecular Probes by Life Technologies	1:500
